# Biodistribution and Trafficking of Hydrogel Nanoparticles in Adult Mosquitoes

**DOI:** 10.1371/journal.pntd.0003745

**Published:** 2015-05-21

**Authors:** Cynthia C. H. Paquette, Yashdeep Phanse, Jillian L. Perry, Irma Sanchez-Vargas, Paul M. Airs, Brendan M. Dunphy, Jing Xu, Jonathan O. Carlson, J. Christopher Luft, Joseph M. DeSimone, Lyric C. Bartholomay, Barry J. Beaty

**Affiliations:** 1 Department of Microbiology, Immunology, and Pathology, Colorado State University, Fort Collins, Colorado, United States of America; 2 Department of Entomology, Iowa State University, Ames, Iowa, United States of America; 3 Lineberger Comprehensive Cancer Center, University of North Carolina at Chapel Hill, Chapel Hill, North Carolina, United States of America; 4 Eshelman School of Pharmacy, University of North Carolina at Chapel Hill, Chapel Hill, North Carolina, United States of America; 5 Department of Chemistry, University of North Carolina at Chapel Hill, Chapel Hill, North Carolina, United States of America; 6 Institute for Nanomedicine and Institute for Advanced Materials, University of North Carolina at Chapel Hill, Chapel Hill, North Carolina, United States of America; 7 Department of Chemical and Biomolecular Engineering, North Carolina State University, Raleigh, North Carolina, United States of America; 8 Sloan-Kettering Institute for Cancer Research, Memorial Sloan-Kettering Cancer Center, New York, New York, United States of America; Liverpool School of Tropical Medicine, UNITED KINGDOM

## Abstract

**Background:**

Nanotechnology offers great potential for molecular genetic investigations and potential control of medically important arthropods. Major advances have been made in mammalian systems to define nanoparticle (NP) characteristics that condition trafficking and biodistribution of NPs in the host. Such information is critical for effective delivery of therapeutics and molecules to cells and organs, but little is known about biodistribution of NPs in mosquitoes.

**Methodology/Principal Findings:**

PRINT technology was used to construct a library of fluorescently labeled hydrogel NPs of defined size, shape, and surface charge. The biodistribution (organ, tissue, and cell tropisms and trafficking kinetics) of positively and negatively charged 200 nm x 200 nm, 80 nm x 320 nm, and 80 nm x 5000 nm NPs was determined in adult *Anopheles gambiae* mosquitoes as a function of the route of challenge (ingestion, injection or contact) using whole body imaging and fluorescence microscopy. Mosquitoes readily ingested NPs in sugar solution. Whole body fluorescence imaging revealed substantial NP accumulation (load) in the alimentary tracts of the adult mosquitoes, with the greatest loads in the diverticula, cardia and foregut. Positively and negatively charged NPs differed in their biodistribution and trafficking. Following oral challenge, negatively charged NPs transited the alimentary tract more rapidly than positively charged NPs. Following contact challenge, negatively charged NPs trafficked more efficiently in alimentary tract tissues. Following parenteral challenge, positively and negatively charged NPs differed in tissue tropisms and trafficking in the hemocoel. Injected NPs were also detected in cardia/foregut, suggesting trafficking of NPs from the hemocoel into the alimentary tract.

**Conclusions/Significance:**

Herein we have developed a tool box of NPs with the biodistribution and tissue tropism characteristics for gene structure/function studies and for delivery of vector lethal cargoes for mosquito control.

## Author Summary

Emerging insecticide resistance in disease vectors is of great public health concern. Discovery of new targets and novel strategies for insecticidal interventions to control vector borne diseases is a public health imperative. Nanotechnology offers great potential for molecular genetic investigations and for delivery of effector molecules for control of disease vectors. We have developed a tool box of hydrogel nanoparticles (NPs) with the biodistribution and tissue tropism characteristics for gene structure/function studies and for delivery of vector lethal cargoes to adult mosquitoes. Nanotechnology will likely be useful for molecular investigations and potential control of the arthropod vectors of other neglected tropical diseases.

## Introduction

Arthropod vectors and pest species are of enormous public health, agricultural, and economic importance. Control of these arthropods is predicated to a large extent on chemical insecticides. Ominously, many vector and pest species have developed or are developing resistance to conventional classes of insecticides. Insecticide resistance is emerging as a major threat for the control of mosquito vectors of human diseases, including *Anopheles gambiae*, the principal vector of malaria in Africa [1–3]. Identification of new targets and development of new approaches for control of vectors is a public health imperative. Improved and efficient techniques to investigate the molecular biology of and to characterize gene structure and function in arthropods would be of great value to identify novel targets for control. New approaches to deliver effector molecules and compounds to improve vector or pest control would also be of great value. Nanotechnology offers great potential in both of these areas. For example improved nanoparticle (NP) delivery of dsRNA to induce RNAi to silence and functionally characterize genes and to cause insect mortality offers exciting new potential for research as well as insect vector and pest management [4, 5].

Physical and chemical properties of natural objects have been refined by nature to optimize biological functions and interactions [6–14]. There are many biological barriers in an organism that condition the efficacy of NP delivery to target tissues and cells [15, 16]. In the human host, these include the vascular endothelium and walls of blood vessels, physical entrapment in organs, phagocytosis, and overall clearance of the NPs from the circulatory system. Physicochemical properties, e.g. size, shape, aspect ratio, modulus, and surface charge, are determinants of the biodistribution and trafficking of NPs *in vivo* in vertebrates and of internalization of NPs into cells [17–21]. The PRINT platform particle technology offers exceptional capability to mimic nature’s handiwork. Particles have been engineered to deliver siRNAs (and other biological molecules) to knock down target gene expression in both *in vitro* and *in vivo* systems [22–25]. The NPs containing the siRNAs are effectively delivered to target cells, where the particle is degraded in the endolysosome, and the siRNAs are then released into the cytoplasm to engage the host RNAi machinery. Major advances have been made in the development and optimization of NPs for delivery of drugs, antigens, and RNAs *in vitro* and *in vivo* in mammalian systems [26–29].

Microencapsulation techniques have been used to enhance the stability, effectiveness and environmental delivery of effector molecules, e.g.—insecticides, to control mosquitoes in nature [30], but development of NP-delivered effector molecules, such as dsRNAs, to control mosquitoes is in its infancy. Chitosan NPs have been used to deliver dsRNA to silence chitin synthase in *An*. *gambiae* mosquitoes [31], and chitosan-siRNA particles have been used to disrupt expression of an olfactory gene in *Ae*. *aegypti* [32]. Other types of NPs have been investigated for vector control [33–36]. The biodistribution of NP-delivered dsRNA to silence a chitinase-like gene in larval *Drosophila melanogaster* has been reported [37]. However, there is little information about NP physicochemical determinants of trafficking of NPs in mosquitoes following environmental (oral or contact) delivery. Following ingestion by adult mosquitoes, NPs would have to traffic in the alimentary tract, penetrate diverticula, cardia, foregut, or midgut barriers, disseminate into the hemocoel and then be internalized by target tissues and cells. Following contact delivery, NPs must traverse cuticular barriers and then traffic in the insect to be internalized by the appropriate tissues and organs. Determination of the optimal physicochemical characteristics of NPs to negotiate these barriers to deliver their cargoes to target cells is the goal of our research.

In this and the accompanying paper [38], we determined the biodistribution and trafficking of poly(ethylene glycol) (PEG) hydrogel particles *in vivo* in adult and larval *An*. *gambiae* mosquitoes following oral, parenteral or contact challenge, and the internalization potential of particles *in vitro* in cell cultures [38]. PRINT technology was utilized to prepare PEG NPs of defined size, shape, aspect ratio, and surface charge for mosquito challenges. In this paper, fluorescently-labeled NPs without cargoes were used to challenge adult *An*. *gambiae* mosquitoes, and the NPs with the preferred biodistribution characteristics (e.g. organ, tissue and cell tropisms and trafficking kinetics) for delivery of molecules to tissues and cells in mosquitoes were identified.

## Methods

### PRINT Nanoparticle Fabrication

#### NP materials

Poly(ethylene glycol) diacrylate (M_n_ 700) (PEG_700_DA), 2-aminoethyl methacrylate hydrochloride (AEM), and diphenyl (2,4,6-trimethylbenzoyl)-phosphine oxide (TPO) were purchased from Sigma-Aldrich. Thermo Scientific Dylight 488, PTFE syringe filters (13 mm membrane, 0.22 μm pore size), sterile water, and methanol were obtained from Fisher Scientific. Conventional filters (2 μm) were purchased from Agilent, polyvinyl alcohol (Mw 2000) (PVOH) was purchased from Acros Organics, and Luvitec (MW 64 kDa) was purchased from BASF. PRINT molds (80 nm x 320 nm, 80 nm x 5000 nm, and 200 nm x 200 nm) were obtained from Liquidia Technologies. Tetraethylene glycol monoacrylate (HP_4_A) was synthesized in-house as previously described [39].

#### NP fabrication methods

The PRINT particle fabrication technique has been described in detail previously [16, 40]. The pre-particle solution was prepared by dissolving 3.5 wt% of the various reactive monomers in methanol. The reactive monomers included: a cure-site monomer (an oligomeric PEG with a nominal molar mass of 700 g/mol terminally functionalized on both end groups with an acryloxy functionality); a hydrophilic monomer used to make up the majority of the particle composition (HP_4_A); an amine containing monomer (AEM) which served to provide a positive charge; and a polymerizable fluorescent tag (Dylight 488). In all cases a photoinitiator, TPO, was also added. Two different pre-particle solutions were used throughout the following studies. For negatively charged particles the pre-particle solution was comprised of 88 wt% HP_4_A, 10 wt% PEG_700_DA, 1 wt% Dylight maleimide 488, and 1 wt% TPO. For positively charged particles, the pre-particle solution was comprised of 68 wt% HP_4_A, 20 wt% AEM, 10 wt% PEG_700_DA, 1 wt% Dylight maleimide 488, and 1 wt% TPO. Using a # 3 Mayer rod (R.D. Specialties), a thin film of the pre-particles solution was drawn onto a roll of freshly corona treated PET, using a custom-made roll-to-roll lab line (Liquidia Technologies) running at 12 ft/min. The solvent was evaporated from this delivery sheet by exposing the film to a hot air dam derived from heat guns. The delivery sheet was laminated (80 PSI, 12 ft/min) to the patterned side of the mold, followed by delamination at the nip. Particles were cured by passing the filled mold through a UV-LED (Phoseon, 395 nm, 3 SCFM N_2_, 12 ft/min). Either a PVOH (for positively charged particles) or a Luvitec (for negatively charged particles) harvesting sheet was hot laminated to the filled mold (140°C, 80 PSI, 12 ft/min). Upon cooling to room temperature, particles were removed from the mold by splitting the harvesting sheet from the mold. Particles were then harvested by dissolving the harvesting film in a bead of water (1 mL of water per 5 ft of harvesting sheet). The particle suspension was passed through a 2 μm filter (Agilent) to remove any large particulates. To remove the excess harvesting material, particles were centrifuged (Eppendorf Centrifuge 5417R) at ca. 21,000 g for 15 min, the supernatant was removed and the particles were re-suspended in sterile water. This purification process was repeated four times.

#### NP physicochemical characterization

**Fig 1:**
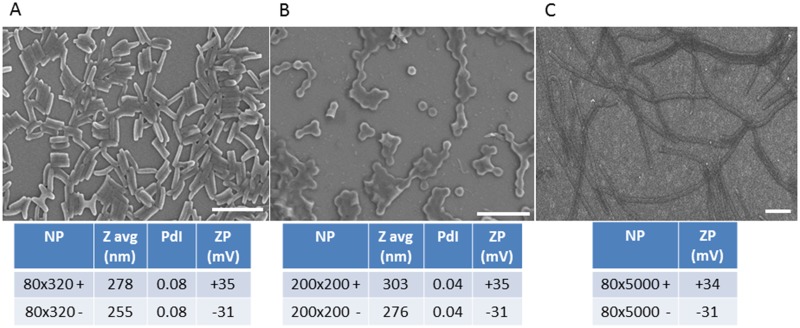
Physicochemical characteristics of nanoparticles manufactured and successfully used to challenge *Anopheles gambiae* mosquitoes. Representative scanning electron micrographs and dynamic light scattering characterization of (A) 80 nm x 320 nm, (B) 200 nm x 200 nm, and (C) 80 nm x 5000 nm PRINT Particles. Bars in micrographs = 1μm. (NP—nanoparticle; Z avg—average hydrodynamic particle diameter; PdI—polydispersity index: ZP—zeta potential)

Stock particle concentrations were determined by thermogravimetric analysis (TGA) using a TA Instruments Q5000 TGA. TGA analysis was conducted by pipetting 20 μL of the stock NP solution into a tarred aluminum sample pan. Samples suspended in water were heated at 30°C/min to 130°C, followed by a 10 min isotherm at 130°C, cooled at 30°C/min to 30°C, followed by a 2 min isotherm at 30°C. TGA was also performed on a 20 μL aliquot of supernatant from a centrifuged sample of the stock solution to account for the mass of any stabilizer remaining in each sample. The concentration of stabilizer was subtracted from the concentration of stock particle solution to determine the actual particle concentration. Particles were visualized by scanning electron microscopy (SEM) using a Hitachi S-4700 SEM (Fig 1). Prior to imaging, SEM samples were coated with 1.5 nm of gold-palladium alloy using a Cressington 108 auto sputter coater. Particle size and zeta potential were measured by dynamic light scattering (DLS) on a Zetasizer Nano ZS (Malvern Instruments, Ltd.) (Fig 1).

### Mosquito Life Cycle

*An*. *gambiae* (G3) strain was used in all experiments, and eggs to start the colony were kindly provided by the Malaria Research and Reference Reagent Resource Center (MR4) (http://www.mr4.org). Rearing and manipulation of mosquitoes generally followed the MR4 recommendations. Briefly, mosquitoes were reared at 27°C (±1°C), 80% (±5%) humidity and a light cycle of 30 min sunrise (at 5:30 AM), 11.5h daylight, and 30 min sunset (at 5:30 PM). Mosquito rearing was conducted in the insectary facilities of the Arthropod-borne and Infectious Diseases Laboratory at Colorado State University.

### NP Biodistribution in Mosquitoes

#### Approach and rationale

The biodistribution and trafficking kinetics of the respective NPs in mosquitoes were determined following oral (ingestion), parenteral (intrathoracic injection) or contact (cuticular) challenges of 5–7 days old (post eclosion) female *An*. *gambiae* mosquitoes. For oral challenge, mosquitoes were exposed to the NPs in 10% sucrose solution for 1, 2 or 3 or more days depending upon the experiment (see below). The multiple day “*ad libitum*” challenges were included to simulate potential multiple feedings at a bait station in nature. The parenteral challenge studies were conducted to determine the biodistribution of NPs in the event that the particles did not escape the midgut or penetrate the cuticular barrier following oral or contact challenge, respectively.

Following challenge, mosquitoes were sacrificed at selected days post challenge and assayed by either 1) whole body fluorescence imaging for determination of NP biodistribution and for fluorescence intensity to estimate NP abundance or loads or 2) by fluorescence microscopic examination of dissected organs tissues, and cells to determine NP tropisms and to estimate NP loads. Mosquitoes were assayed at 0, 1, 3, and 7 days post challenge to determine the trafficking and temporal kinetics of NP biodistribution.

#### Whole body imaging for determination of NP biodistribution and trafficking

The gross anatomic biodistribution and relative NP loads in adult *An*. *gambiae* was determined following parenteral and oral challenge. For parenteral challenge, 1 μg (5 mg/mL) NPs were injected per mosquito in 200 nL volume and images collected at day 0, 1, 3 and 7 post-injection. For oral challenge, the positively or negatively charged NPs were mixed with 10% sucrose resulting in a 1 mg/mL NP solution. Cartons of sugar (24 hr) and water (3 hr) deprived adults (n = 15 per carton) were exposed to 200 μL of this solution on parafilm for 1 or 2 d or *ad libitum* for 7 d without replenishing the NP solution; a thin transparent plastic film was used to cover the cartons to reduce the rate of evaporation. The NPs remained in solution for at least 3–4 d. After 24 hr of NP exposure, sucrose pads were added to the cartons for the duration of the experiment. Mosquitoes were sacrificed and imaged at day 1, 2, 3 and 7 post-initial exposure. Each day, the mosquitoes to be imaged were immobilized by freezing at -20°C (~5 min), and legs and wings were removed from bodies, which were placed on the imaging tray.

Images were captured using an In Vivo Multispectral FX Pro imaging systems (Carestream) using 480 nm excitation and 535 nm emission wavelengths. Fluorescence exposure conditions were kept the same throughout the experiment. Mean Fluorescent Intensity (MFI) values were calculated using NIH Image Jv1.47m. A white light image was also captured to define the mosquito boundaries and draw regions of interest (ROI). The same ROI was then applied on the fluorescent image and the mean fluorescence was quantified using the *Analyze→Measure function*. In order to correct the differences between inherent fluorescent intensities between different NP groups, a correction factor was applied to the raw MFI. The correction factor was defined as the ratio of fluorescent intensity of the NP group to the intensity value of the brightest NP group (80 nm x 320 nm negatively charged). The resulting number was then divided by the raw MFI obtained for each individual. Statistical analysis was performed using JMP® software (SAS Institute). Data were log transformed and comparisons between treatments were made by Tukey's HSD (honest significant difference). Differences were considered significant for p < 0.05.

#### Microscopic determination of NP organ, tissue, and cell tropisms, loads, and trafficking

Studies were conducted to characterize the biodistribution of NPs following oral, parenteral, and contact challenges.

For oral challenges, mosquitoes were exposed to the respective NPs in 10% sucrose solution for 1 or 3d. For each challenge, 40 *An*. *gambiae* female mosquitoes were placed in 16 cup square food storage containers (Mainstay, Walmart) with netting and sleeve, sugar (12 hr) and water (3 hr) deprived and then exposed to the respective NP and sucrose suspension for 1 or 3 d. For the 1 d challenge, 600 μL of either 250 μg/mL or 50 μg/mL (high dose and low dose, respectively) of the positively or negatively charged 80 nm x 320 nm, 200 nm x 200 nm or 80 nm x 5000 nm hydrogel particles were dispensed in a cap of a 15 mL conical tube and placed in the respective cups. After 1 d, the caps were removed, and mosquitoes were provided water and sugar solution *ad libitum*. For the 3 d challenges, mosquitoes were allowed to feed *ad libitum* on a suspension of 80 nm x 320 nm NPs for 3 d. NP solutions were replenished daily. For each of the challenge groups, five mosquitoes were collected at 1, 2, 4 and 7 d post eclosion for determination of NP biodistribution by fluorescence microscopy.

For parenteral challenges, mosquitoes were intrathoracically injected with the respective NPs. Female mosquitoes were sugar (12 hr) and water (3 hr) deprived prior to injection. Mosquitoes were parenterally challenged with approximately 1μL of the respective NP suspension (250 μg/mL) using either a syringe injector or with 69 nL of the NP suspensions using a Nanoject II injector (Drummond Scientific Company). Control mosquitoes were injected with *Anopheles* saline. After injection, mosquitoes were maintained in 16 oz Solo food containers in the insectary, provided 10% sugar water *ad libitum*. Five females were dissected at 1 and 2 d post injection to assay for NP biodistribution by fluorescence microscopy.

**Fig 2:**
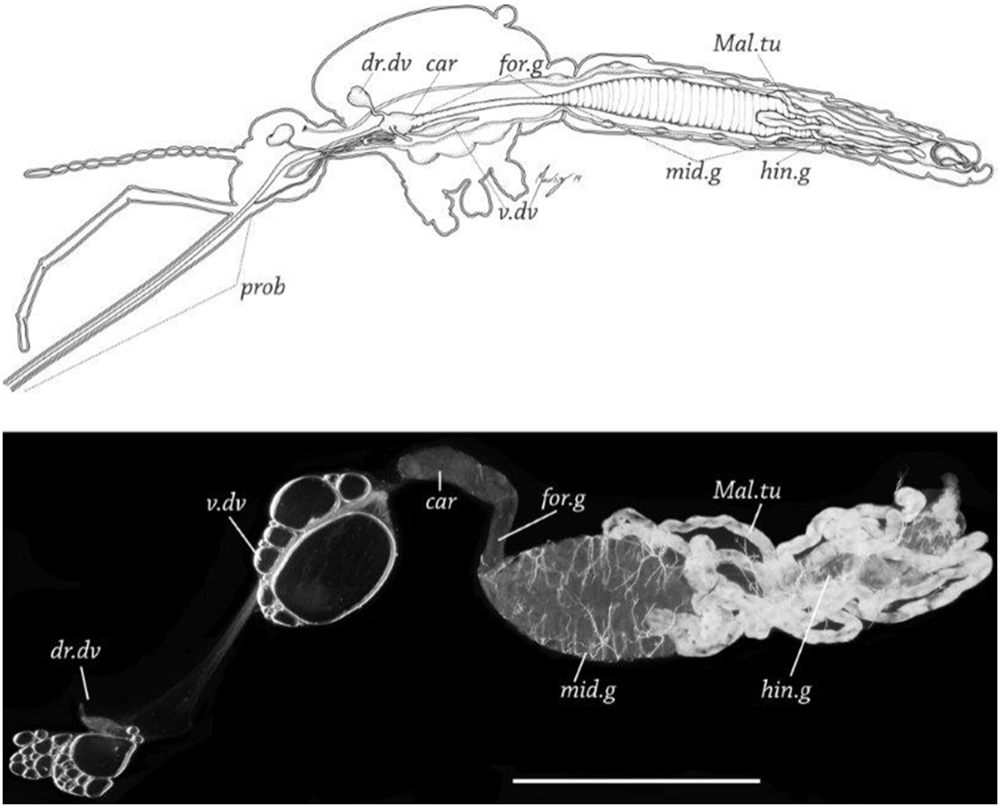
*Anopheles gambaie* adult female alimentary tract and dissected principal organ systems. A. Sagittal plane diagram of organ systems *in situ* in *An*. *gambiae*. Illustration by R. Isaì Madriz. B. Dissected alimentary tract from female *An*. *gambiae*. prob = proboscis,dr. dv = dorsal diverticulum, v. dv = ventral diverticulum,. car = cardia, for.g = foregut, mid.g = midgut, hin.g = hind gut, Mal.tu = Malpighian tubules, Bar = 500 μM.

For contact challenges, a 69 nL drop of fluorescently labeled 80 nm x 320 nm positively or negatively charged NPs (3.6 μg/mL) in water or NP40 (nonyl phenoxypolyethoxylethanol) (0.5%) was placed on of the head/proboscis, thorax or abdomen of adult female mosquitoes (5 to 7 d post-eclosion) using the Nanoject II injector system. All deliveries were performed using a dissecting microscope using glass needles prepared using a vertical pipette puller (P-30, Sutter Instrument Co.). To determine NP tropisms, mosquito tissues were examined for the presence of fluorescence 1 and 2 d post contact; the alimentary tract was dissected and the thoraces, heads, and abdomens were squashed on glass slides (Fig 2), and assayed for NP biodistribution by fluorescence microscopy.

### Fluorescence Microscopy

To determine organ, tissue and cell tropisms and to estimate the abundance (load) of the respective NPs, alimentary tract tissues (ventral diverticulum, dorsal diverticula, foregut, midgut, hindgut, and Malpighian tubules) and non-alimentary tract tissues (head, proboscis, salivary glands, thoracic muscles, ovaries, and tracheae) were dissected from mosquitoes (Fig 2) at predetermined days post challenge. Organs and tissues were mounted on slides, covered with PBS-10% glycerol mounting medium, and assayed for the presence and abundance of NPs in tissues up to 7 d post challenge using an Olympus BH2-RFCA fluorescence microscope or a Leica DM4500B fluorescence microscope. NP tropisms were determined by detection of any level of fluorescence signal (1 to 5+) in the respective organ, tissue, or cell. The intensity of the fluorescent signal was subjectively scored from 0 to 5+. NP loads in the respective organ or tissue were assumed to be directly correlated with the relative intensity of the fluorescent signal in the organ or tissue.

In some studies, to more accurately determine cell tropisms of selected NPs, alimentary tract tissues were dissected in PBS and fixed in 4% formaldehyde (Electron Microscopy) for at least 1 hr at room temperature (RT). The fixative was then removed, the tissues were rinsed three times with PBS 5 min each at RT and then permeabilized in 1% Triton X-100 for 10 min at RT and then washed three times for 5 min each at RT. The tracts were then incubated in a 1:40 dilution of—Alexa Fluor 546 phalloidin (high-affinity F-actin probe conjugated to bright, photo stable, orange-fluorescent Alexa Fluor 546, lifetechnologies.com/ product A22283) for 15 min at RT. After rinsing with PBS (three times for 5 min each at RT), alimentary tracts were placed onto slides, covered with Vectashield (Vector Laboratories Inc., Burlingame, CA), and a coverslip was sealed with nail polish on the slide. Biodistribution of NPs was determined using a Leica DM4500B fluorescence microscope.

## Results

### NP Effect on Challenged Adult *An*. *gambiae*

Female mosquitoes were exposed to the respective NPs (Fig 1) in 10% sucrose (250 μg/mL and 50 μg/mL) for 1 d and then assayed by image analysis and immunofluorescence microscopy for NP biodistribution, trafficking, and kinetics of tissue tropisms. Nearly 100% of *An*. *gambiae* females readily ingested the NP-sucrose meals regardless of particle size and charge. NP ingestion exhibited little untoward effect on the mosquitoes; for example, in a typical experiment, the survivorship rates for mosquitoes ingesting positively (N = 30) or negatively (N = 30) charged 80 nm x 320 nm NPs (250 μg/mL) or sucrose (N = 28) were 93, 100, and 71%, respectively. Similarly, parenteral challenge of larval mosquitoes or *in vitro* challenge of mosquito cells in culture (see Figs 6 and 7 in companion paper [38]) caused little differences in larval survivorship or cell viability.

### Biodistribution of NPs in Whole Bodies of Mosquitoes Following Parenteral and Oral Challenge

**Fig 3:**
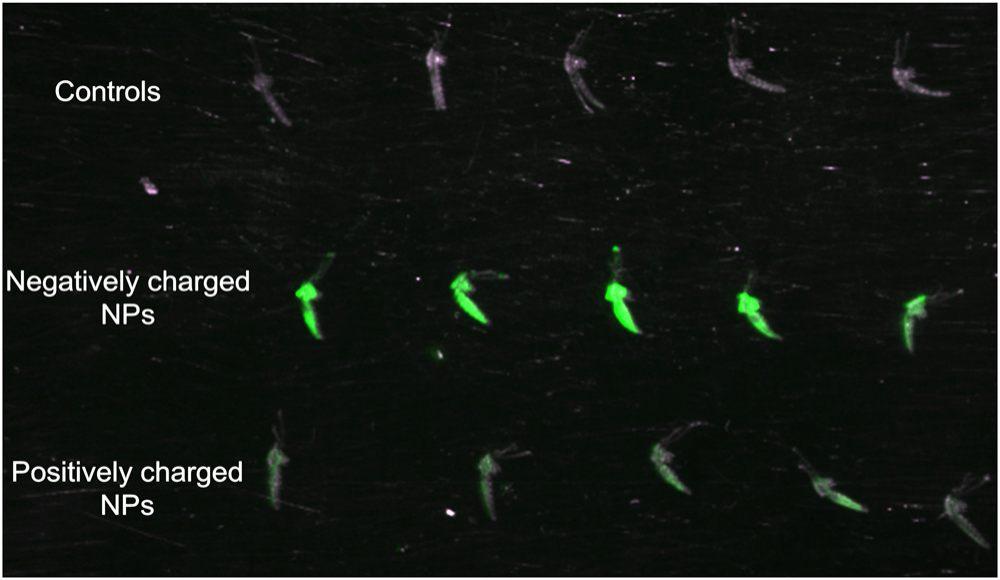
Whole body imaging of *Anopheles gambiae* females parenterally challenged with 200 nm x 200 nm hydrogel nanoparticles. Mosquitoes were sacrificed and imaged 1 day post challenge. Fluorescence signal was detected in the thorax and abdomen of NP challenged mosquitoes and was visibly brighter for females challenged with negatively charged NPs (row 2) compared to those challenged with positively charged particles (row 3). Fluorescence signal was not detected in non-challenged control mosquitoes (row 1).

**Fig 4:**
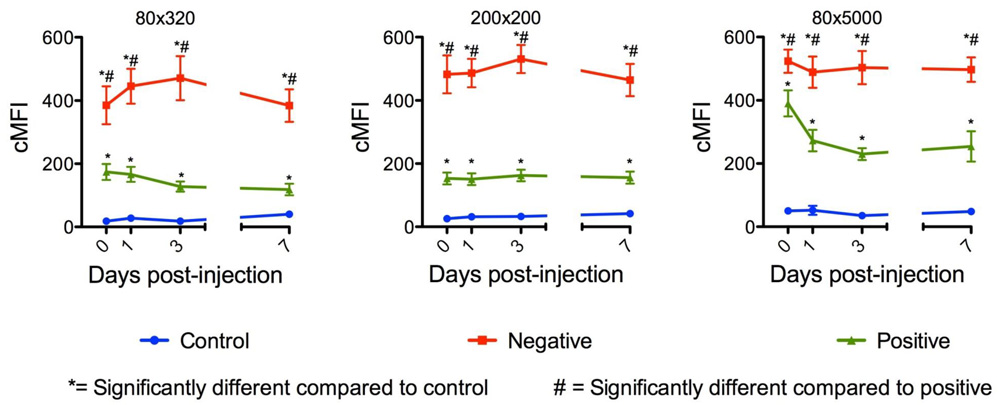
Whole body mean fluorescence intensity (MFI) values of *Anopheles gambiae* females parenterally challenged with 80nm x 320 nm, 200 nm x 200 nm and 80 nm x 5000 nm hydrogel nanoparticles. Negatively charged NPs consistently produced MFI values that were significantly higher than positively charged NPs.

Whole body image analysis was used to detect and quantify NPs following parenteral and oral challenges. Adults were injected with positively or negatively charged 200 nm x 200 nm NPs (250 μg/mL) or sucrose. At 1 d post challenge, mosquitoes injected with the negatively charged NPs exhibited the greater fluorescent signal (Fig 3, Row 2). The greatest Mean Fluorescence Intensity (MFI) values were also detected in mosquitoes challenged with negatively charged NPs (Fig 4), and the MFI values at day seven were similar to those at the day of challenge, with the exception of the 80 nm x 5000 nm positively charged NPs (Fig 4). The reasons for the dramatic differences in MFI between positively and negatively charged NPs following parenteral challenge (Fig 4) may be attributable to more efficient internalization of the positively charged NPs [38].

**Fig 5:**
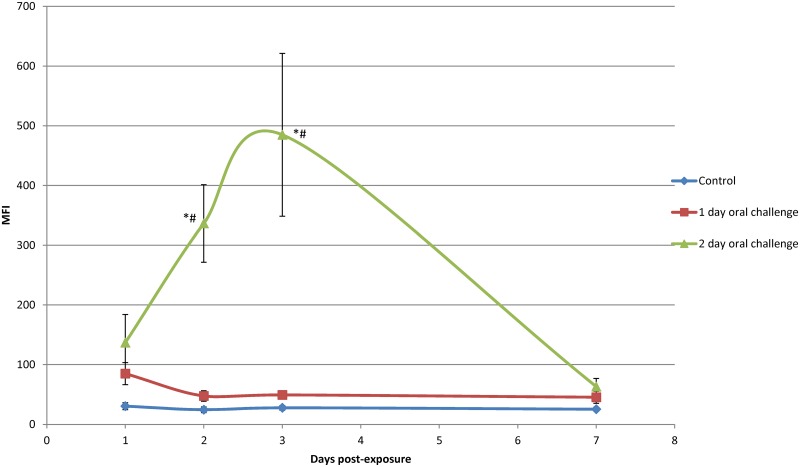
Whole body mean fluorescence intensity (MFI) values of *Anopheles gambiae* females orally challenged with negatively charged 200 nm x 200 nm hydrogel particles for 1 and 2 day(s). MFI values for females orally challenged for 1 d with NPs were low compared to MFI values for females orally challenged for 2 d in which MFI values increased and peaked at approximately 3 d post challenge and decreased thereafter. Statistical difference (p<0.05) from controls and 1 day oral challenge groups are indicated by * and #.

**Fig 6:**
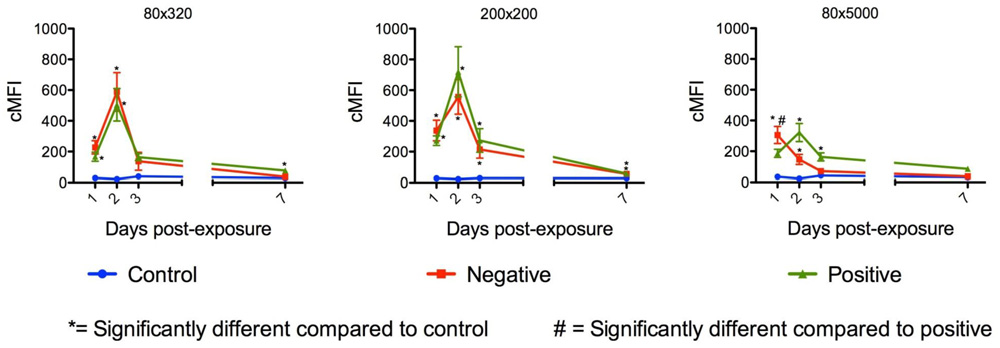
Whole body mean fluorescence intensity (MFI) values of *Anopheles gambiae* females orally challenged *ad libitum* with positively and negatively charged 80nm x 320 nm, 200 nm x 200 nm and 80 nm x 5000 nm nanoparticles. MFI values for females orally challenged with 80 nm x 320 nm and 200 nm x 200 nm NPs showed similar patterns over time with an increase in MFI values the first two days of challenge and a decrease thereafter. However, MFI values for females orally challenged with 80 nm x 5000 nm NPs showed a different pattern with lower MFI values and the MFI values for negatively charged NPs declined after 1 d post oral challenge.

Oral challenge of mosquitoes yielded different results. Following oral challenge with 200 nm x 200 nm negatively charged NPs for 1 d, MFI values remained very low (Fig 5). However, following oral challenge for 2 d, MFI values increased significantly to 3 d post exposure and then declined thereafter (Fig 5). These results were confirmed by oral challenge of mosquitoes with positively and negatively charged 80 nm x 5000 nm and 200 nm x 200 nm NPs (Fig 6), which also increased in abundance following *ad libitum* oral challenge. The MFI indices declined to essentially undetectable levels at day seven post-initial exposure. The positively charged, but not the negatively charged, 80 nm x 5000 nm NPs exhibited a similar pattern (Fig 6).

#### Biodistribution and trafficking of NPs s in organs, tissues, and cells following 1 d oral challenge

**Fig 7:**
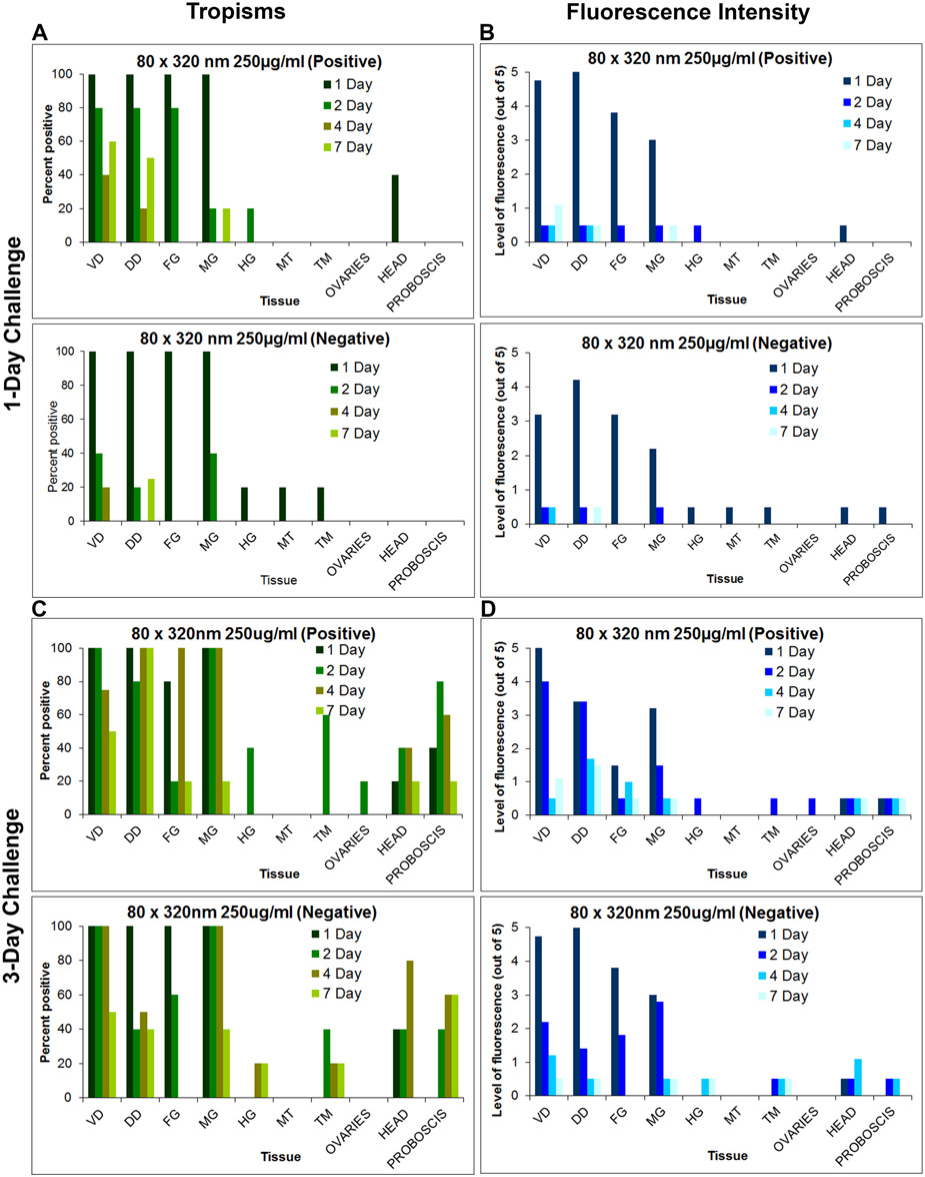
Tissue tropisms and fluorescence intensity of positively and negatively charged 80 nm x 320 nm hydrogel nanoparticles in *Anopheles gambiae* females following 1 and 3 day(s) oral challenges. Tissue tropisms (percent of mosquitoes with NP fluorescent signal of any intensity detected in the respective organ or tissue) of positively and negatively charged 80 x 320nm NPs in *An*. *gambiae* following 1 day (A) and 3 day (C) oral challenge (250 μg/mL). Fluorescence intensity (mean level of fluorescence intensity (NP load) in organs and tissues containing NPs) of positively and negatively charged 80 x 320 nm NPs in *An*. *gambiae* following 1 day (B) and 3 day (D) oral challenge. In mosquitoes challenged for 1 d, tissue tropisms and fluorescence intensity decreased substantially by 1 or 2 d post challenge. However, some organs/tissues still contained fluorescence signal at 7 d post challenge. In mosquitoes challenged for 3 d, tissue tropisms and fluorescence intensity were greater and of longer duration than in mosquitoes challenged for only 1 d. VD = ventral diverticulum; DD = dorsal diverticula; FG = foregut; MG = midgut; HG = hindgut; MT = Malpighian tubules; TM = thoracic muscles.

**Fig 8:**
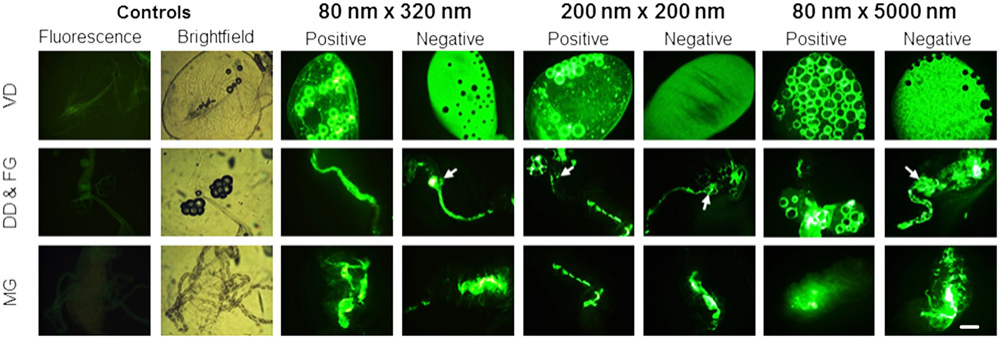
Biodistribution of hydrogel nanoparticles in the alimentary tract of *Anopheles gambiae* females following a 1 day oral challenge. Following oral challenge with the respective NPs (250 μg/mL), fluorescence signal was detected in all alimentary tract tissues, including Ventral diverticulum (VD), dorsal diverticulum (DD), foregut (FG), midgut (MG) and cardia (arrow). Fluorescence was not detected in the alimentary tracts of the control, sucrose-fed females. Bright field micrographs show the outline of the tissues from control mosquitoes that were not challenged with NPs. Bar = 1 μm

To better understand the biodistribution of NPs at the organ and cellular levels over time, 1 d oral challenges were conducted with the respective NPs (Fig 1). Mosquitoes were dissected (Fig 2) and examined using fluorescence microscopy to determine tissue tropisms and the duration of the tropisms (Fig 7A), and the intensity of the NP fluorescent signal overtime (Fig 7B). All NPs exhibited similar alimentary tract tropisms (Fig 8). Following oral challenge with the 250 μg/mL NPs, both positively and negatively charged NPs were detected abundantly in dorsal and ventral diverticula, cardia/foregut, and midgut (Fig 8). Interestingly, large accumulations of both positively and negatively charged NPs were consistently detected at 1 d post challenge in the cardia and foregut (Fig 8), organs which contain the first likely target cells in the alimentary tract that the NPs would encounter following ingestion. The tissue tropisms and intensity of fluorescence were similar for the different NPs. For example, when orally challenged with the 250 μg/mL dose of 80 nm x 320 nm positively and negatively charged NPs, tissue tropisms were greatest on days 1 and 2 post ingestion (Fig 7A). Tissue tropisms and fluorescent intensity then typically declined in organs (confirming the whole body imaging results), and NPs were only detectable in some tissues at low intensity at 7 d post ingestion (Fig 7A and 7B). When the mosquitoes were orally challenged with the 50 μg/mL dose of positively charged 80 nm x 320 nm NPs, tissue tropisms were similar to but of shorter duration (S1A Fig) than in mosquitoes challenged with the 250 μg/mL dose of NPs (Fig 7A). In addition, the fluorescent signal was less intense in mosquitoes challenged with the lower dose of NPs (S1B Fig) than in the mosquitoes challenged with the higher dose of NPs (Fig 7B). The differences in tissue tropisms and fluorescent intensities were not as pronounced when mosquitoes were orally challenged with 250 or 50 μg/mL of negatively charged 80 nm x 320 nm NPs (Figs 7A, 7B, S1A and S1B).

When mosquitoes were orally challenged with 200 nm x 200 nm NPs (250 μg/mL), the tissue tropisms and fluorescence intensities (S2 Fig) were similar to those detected with the 80 nm x 320 nm NPs (Fig 7A and 7B). When mosquitoes were challenged with the 50 μg/mL dose of positively charged 200 nm x 200 nm NPs, tissue tropisms and fluorescence intensity (S2C and S2D Fig) were reduced compared to mosquitoes challenged with the 250 μg/mL dose (S2A and S2B Fig). The reduction in tissue tropisms and fluorescence intensity were not observed in mosquitoes challenged with the 50 μg/mL dose of negatively charged 200 nm x 200 nm NPs (S2C and S2D Fig). Rather, there was an increase in tissue tropisms and duration in the mosquitoes challenged with the lower dose of the NPs (S2A and S2C Fig).

When mosquitoes were orally challenged with 80 nm x 5000 nm NPs (250 μg/mL), the biodistribution pattern (S3 Fig) was similar to that seen following challenge with other NPs (Figs 7A, 7B, S2A and S2B). When mosquitoes were challenged with the lower dose (50 μg/mL) of positively charged 80 nm x 5000 nm NPs, tissue tropisms and fluorescence intensities were reduced (S3C and S3D Fig) in comparison to the mosquitoes challenged with high dose of the NPs (S3A and S3B Fig). When mosquitoes were challenged with the lower dose of negatively charged 80 nm x 5000 nm NPs, the biodistribution pattern was similar to that seen with 200 nm x 200 nm negatively charged NPs (S2A and S2C Fig). There was an increase in tissue tropisms and fluorescent intensities in alimentary tract tissues as compared to mosquitoes challenged with the higher dose of negatively charged 80 nm x 5000 nm NPs (S3A and S3B Fig). The reasons for the different biodistribution patterns and trends between positively and negatively charged particles following low dose challenges remain to be determined.

Overall, there was minimal dissemination of NPs from the alimentary tract into the hemocoel following oral challenge for 1 d; NPs were detected in only a few hemocoel associated tissues, most frequently in the head, thoracic muscle, and proboscis (Figs 7A, S2A, S2C, S3A and S3C).

**Fig 9:**
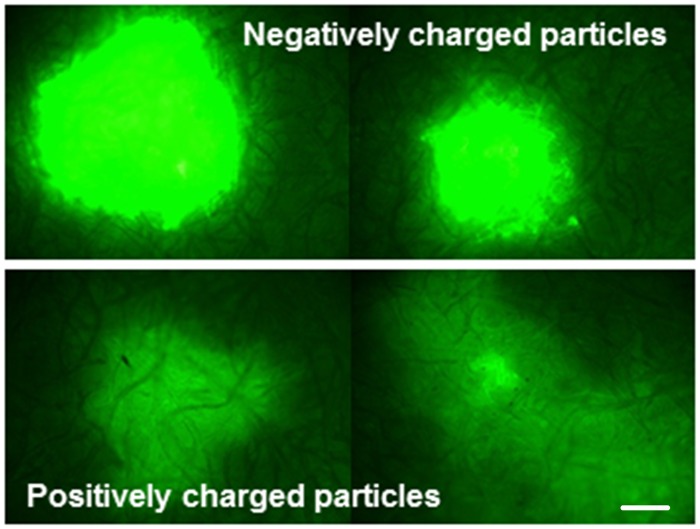
Excretion of 80 nm x 320 nm hydrogel nanoparticles from the alimentary tract of *Anopheles gambiae* females following a 1 d oral challenge. Excretion of negatively charged NPs resulted in larger and brighter fluorescence spots on filter paper lining the cages than excreted positively charged NPs. Bar = 1 μm.

**Fig 10:**
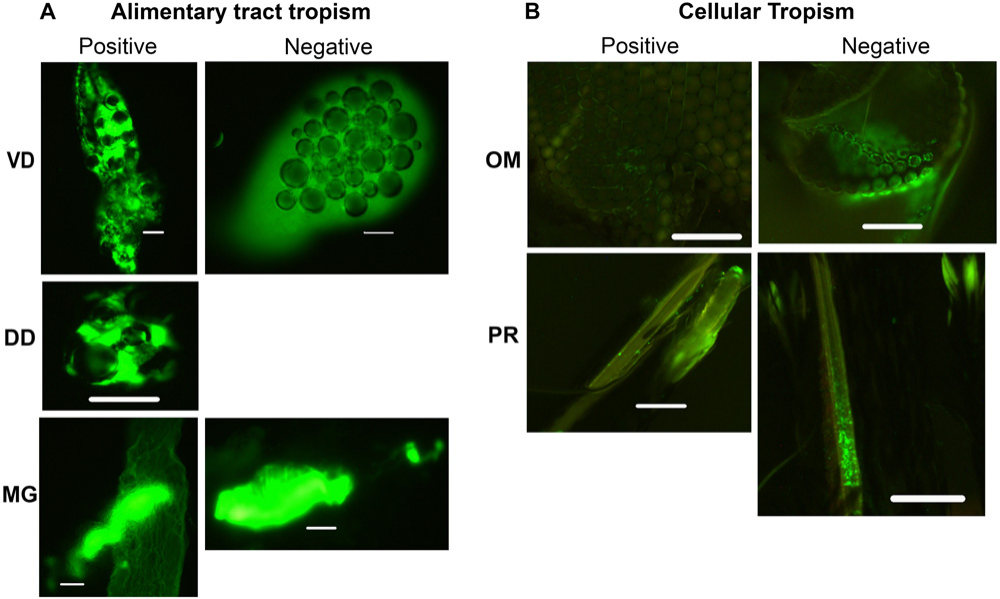
Biodistribution of hydrogel nanoparticles in tissues of *Anopheles gambiae* females following a 3 d oral challenge. (A) Alimentary tract tropisms: Fluorescence was present in many tissues in alimentary tract tissues and organs including the ventral diverticulum (VD), dorsal diverticula (DD), and MG (midgut). (B) Hemocoel associated tropisms: Fluorescent signal was present in tissues associated the hemocoel including ommatidia (OM) and proboscis. Bar = 1 μm

Following oral challenge, the negatively charged NPs apparently transited the alimentary tract more quickly than positively charged NPs; The NPs were detected in fewer tissues than positively charged particles at 2 d post challenge (Fig 7A). To investigate this, filter papers were placed on the bottom of selected cages of mosquitoes challenged with positively or negatively charged NPs to capture expelled fluids. Fluorescence intensity and sizes of spots of NPs expelled during or shortly after ingestion were typically much greater for the negatively charged NPs (Fig 9). Much smaller fluorescent spots were detected on the papers and netting (S4 Fig), which were likely due to physical tracking of NPs on tarsi or the proboscis from the NP feeding suspensions. In this regard, NPs were associated frequently and sometimes abundantly with tissues in the proboscis (Fig 10B), which could account for the smaller fluorescent spots (S4 Fig).

#### Biodistribution and trafficking of NPs in organs, tissues, and cells following 3 d oral challenge

*An*. *gambiae* females were exposed to positively and negatively charged 80 nm x 320 nm NPs for 3 d and then assayed at predetermined days for the presence of NPs and intensity of fluorescence (Fig 7C and 7D). The intensity of the signal, the number of tissues involved, and the duration of the signal were significantly greater than when the mosquitoes were challenged for just 1 d (Fig 7A–7D). NPs were detected in the diverticula, cardia, foregut, and midgut lumen, similar to the tropisms detected following 1 d challenge (Fig 10A). Importantly, both positively and negatively charged NPs were detected in or associated with tissues outside of the alimentary tract following 3 d oral challenge, including head, ommatidia, and proboscis (Fig 10B). Clearly, the NPs had trafficked into the hemocoel. The NP tissue tropisms and loads were greater in alimentary tract organs and tissues in the 3 d *ad libitum* challenged mosquitoes than in the 1 d challenged mosquitoes (Figs 7A–7D and 10), which could condition dissemination of the NPs into the hemocoel.

#### Biodistribution and trafficking of NPs in organs, tissues and cells following parenteral challenge

**Fig 11:**
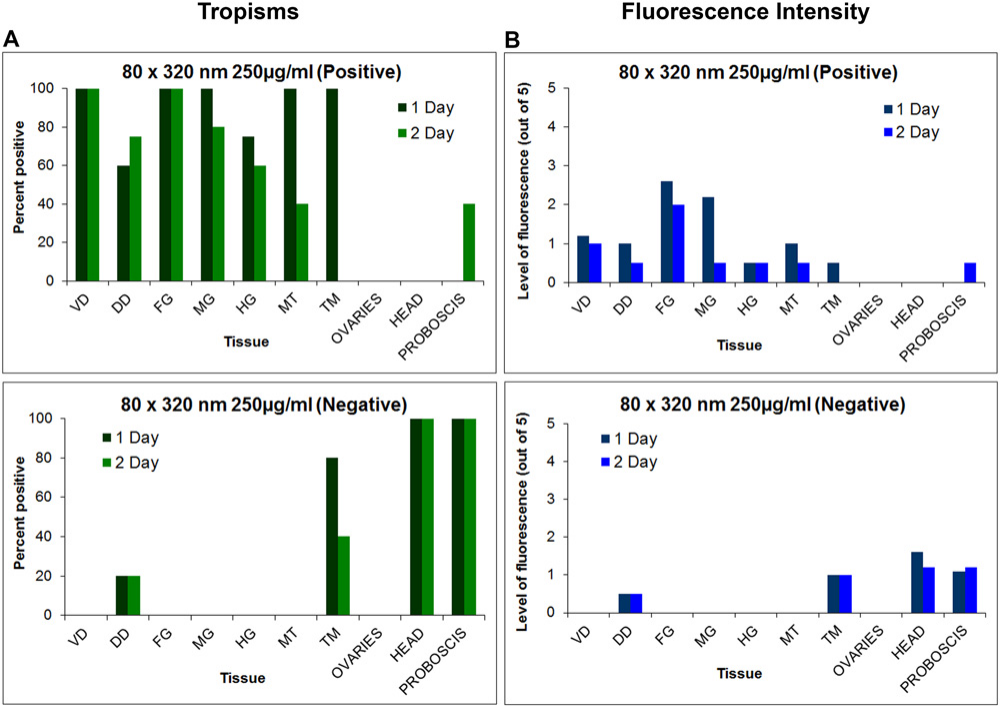
Tissue tropisms and fluorescence intensity of positively and negatively charged 80 nm x 320 nm hydrogel nanoparticles in *Anopheles gambiae* females following parenteral challenge. (A) Tissue tropisms (percent of mosquitoes with NP fluorescent signal of any intensity detected in the respective organ or tissue) of positively and negatively charged 80 x 320nm NPs in *An*. *gambiae* following parenteral challenge (250 μg/ml). (B) Fluorescence intensity (mean level of fluorescence intensity (NP load) in organs and tissues containing NPs) of positively and negatively charged 80 x 320 nm NPs in *An*. *gambiae* organs and tissues following parenteral challenge. NP tissue tropisms and loads were greater and of longer duration in most tissues females injected with positively charged NPs than in those injected with negatively charged NPs. Negatively charged NPs were most frequently associated with head and proboscis tissues. VD = ventral diverticulum; DD = dorsal diverticula; FG = foregut; MG = midgut; HG = hindgut; MT = Malpighian tubules; TM = thoracic muscle.

**Fig 12:**
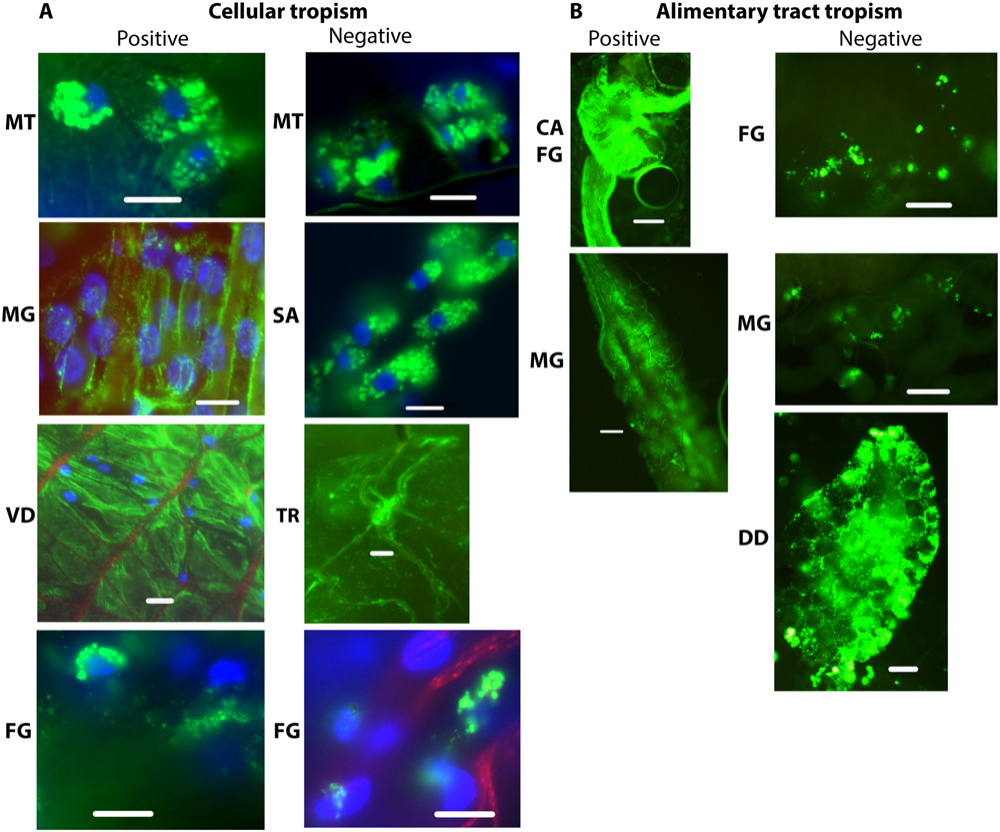
Biodistribution of positively and negatively charged 80 nm x 320 nm hydrogel particles in *Anopheles gambiae* mosquitoes 1 d following parenteral challenge. (A) NP biodistribution in cells and tissues associated with the hemocoel: NPs were detected in or associated with many tissues following parenteral challenge, including Malpighian tubules (MT), salivary glands (SA), and trachea (TR) associated tissues, and in tissues on the surface of organs exposed to the hemocoele such as ventral diverticulum (VD), foregut (FG), and midgut (MG). (B) NP detection in alimentary tract associated organs and tissues: NPs also disseminated from the hemocoele into the alimentary tract and were detected in cardia (CA), foregut (FG), midgut (MG), ventral diverticulm (VD) and dorsal diverticula (DD). Bar = 1 μm

At 1 and 2 d post injection, positively charged NPs were detected in more alimentary tract organs and tissues and at greater intensity than negatively charged NPs (Fig 11). Negatively charged NPs were more associated with head and proboscis tissues (Fig 11A). NPs were detected consistently in and/or associated with multiple organs and cells, including trachea (Fig 12A), muscle and nerves on the surface of organs such as midgut and ventral diverticulum and Malpighian tubules (Fig 12A), and less frequently in hemocytes, head, proboscis, and thoracic muscles. Positively charged NPs were detected in Malpighian tubules in 100% of mosquitoes following parenteral challenge (Figs 11A and 12A), but not in Malpighian tubules following oral challenge (Fig 7A). This suggests that Malpighian tubules take up particles from the hemolymph but not from the alimentary tract. Negatively charged NPs were very infrequently detected in Malpighian tubules following oral challenge (Fig 7A), and not detected following parenteral challenge (Figs 7A and 11A). Negatively charged NPs were frequently detected abundantly in tissues of the proboscis and head, a similar biodistribution to that demonstrated for negatively charged NPs following 3 d *ad libitum* challenge (Fig 10B). In general, following injection, negatively charged NPs were more associated with punctate fluorescence, and positively charged NPs were more associated with the basal lamina of multiple organs (Fig 12B) Following injection, positively charged NPs persisted in organs and tissues longer or were more stable (detectable over time) than negatively charged NPs (Fig 11). Negatively charged NPs are probably not internalized by the cells in tissues and organs and released with the hemolymph upon dissection (see Fig 1 in companion paper [38]).

Following injection, both positively and negatively charged NPs trafficked into the alimentary tract of mosquitoes and were detected frequently in alimentary tract tissues, including the ventral and dorsal diverticula, foregut, midgut, and hindgut (Fig 12B). NPs were detected intracellularly in alimentary tract cells following injection, including the foregut and midgut (Fig 12B).

### Biodistribution and Trafficking of NPs in Organs, Tissues, and Cells Following Contact Challenge

**Fig 13:**
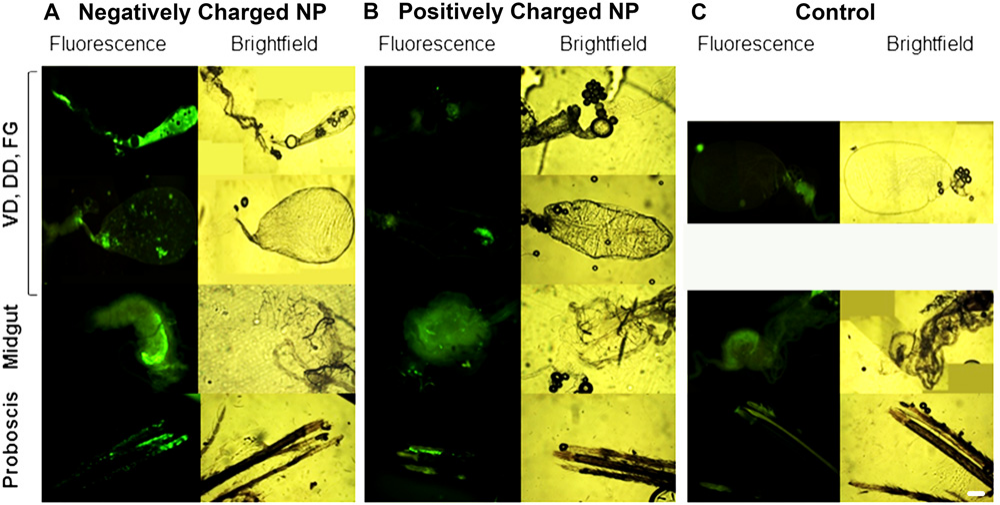
Biodistribution of 80 nm x 320 nm hydrogel nanoparticles in the alimentary tracts of *Anopheles gambiae* females 1 d following contact challenge. Female mosquitoes were challenged by administration of NPs to the head. (A) Negatively charged NPs. (B) Positively charged NPs. (C) Control mosquitoes. Negatively charged NPs trafficked more effectively and were detected more frequently and abundantly in alimentary tract tissues and the proboscis than positively charged NPs. VD = ventral diverticulum; DD = dorsal diverticula; FG = foregut; Bar = 1 μm

Adult mosquitoes were challenged by contact with positively or negatively charged 80 nm x 320 nm NPs. A drop of the respective particle solution with NP40 (0.5%) was placed on the head, thorax, or abdomen. Mosquitoes were dissected at 1 or 2 d post challenge and tissues were examined for fluorescence. Administration of negatively charged NPs to the head resulted in NP detection in head tissues, proboscis and alimentary tract tissues, including diverticula and foregut) in 100% (5/5) of mosquitoes 1 d post challenge (Fig 13A). Fluorescence signal was detected in midgut tissue of 60% (3/5) mosquitoes at 2 d post challenge, but minimal signal was detected in the diverticula at that time. Signal remained intense in certain tissues in the proboscis 2 d post challenge. Following contact challenge to the head with positively charged 80 nm x 320 nm NPs, fluorescence signal was detected in 40% (4/10) of mosquitoes in the alimentary tract tissues and in the proboscis; however the fluorescent signal was much less intense (Fig 13B). than that detected in mosquitoes challenged with the negatively charged NPs (Fig 13A). Contact challenges with positively or negatively charged NPs to the thorax or abdomen were not promising. Following administration of positively or negatively charged NPs to the thorax, very minimal fluorescent signal was detected at 1 or 2 d post challenge in the alimentary tract or proboscis of 43% (12/28) of the challenged mosquitoes. Following administration of positively or negatively charged NPs to the abdomen, very minimal fluorescent signal was detected in tissues of 22% (4/18) of the challenged mosquitoes. The fluorescence signal was typically scored as <1 (on the scale of 0–5+), and it was difficult to differentiate the mosquitoes challenged by these routes from the control mosquitoes (Fig 13C). Clearly trafficking of NPs into mosquitoes following throrax or abdomen contact challenges is inefficient.

## Discussion

The studies provided important information for the potential use of and preferred physical characteristics of hydrogel NPs for delivery of cargoes (e.g. dsRNA) to silence mosquito genes for functional analyses and for mosquito control. Both adult and larval mosquitoes [38] readily ingested the NPs tested. Following 1 d oral challenge of adults positively and negatively charged NPs were detected in dorsal and ventral diverticula, cardia, foregut, and midgut at day one post ingestion (Fig 7). By day two post ingestion, signal decreased, especially for negatively charged NPs in both the number of tissues exhibiting fluorescence and in fluorescence intensity (Fig 7). When orally challenged for 1 d, there was minimal detection of the respective NPs in non-alimentary tract tissues (Fig 7). However, when mosquitoes were challenged for 2 d or more, the biodistribution of NPs changed dramatically (Figs 5, 6 and 7). Multiple day challenges resulted in dramatic increases in MFI values, which were nearly undetectable in whole body images of mosquitoes that were orally challenged for just 1 d (Fig 5). The reason for this is unclear. The NP load is clearly increased in the mosquitoes challenged for multiple days (Fig 5), and both positively and negatively charged NPs are detected abundantly and persist in the alimentary tract (Fig 7). Multiple day challenges also results in NP dissemination to non-alimentary tract tissues (Fig 7), and the resulting biodistribution of NPs was similar to that seen with NPs following parenteral challenge where NPs were detected in or associated with tracheae and tracheoles, cardia, proboscis, etc. (Fig 12). The decline in MFI values beginning at day two (Figs 5 and 6) was unexpected. Sugar pads were placed on the cages after 1 d, and perhaps mosquitoes began to feed preferentially on the sugar pads.

The anatomic basis for trafficking of NPs out of the alimentary tract remains to be determined. Multiple ingestions of NPs may somehow perturb tissue barriers and promote accumulating and trafficking of NPs. However, trafficking of NPs from the hemocoel into the alimentary tract also occurs following parenteral challenge; NPs were detected in the cardia, foregut, and midgut of mosquitoes (Fig 12B). The large accumulation of particles in the cardia and foregut (Figs 8 and 12) following oral or parenteral challenge suggests that this organ may be involved in trafficking. Arboviruses, which must disseminate from the vector gut to infect salivary glands to be transmitted, have been detected in the cardia of infected mosquitoes. Investigators have proposed that the intussusception of the foregut and esophagus, which may be only a cell or two thick, is a likely anatomic mechanism for arbovirus dissemination [41]. The abundant accumulation of NPs in the cardia could result in trafficking of the particles between the body compartments. Arboviruses can also traffic from the midgut into the hemocoel via tracheae [42, 43]. The association of NPs with tracheae (Fig 12A) is also provocative in this regard. The exact mechanism conditioning the trafficking remains to be determined.

There were major differences in the biodistribution and trafficking of positively and negatively charged NPs. Following parenteral challenge, positively charged NPs seemed to coat the basal lamina of multiple organs; negatively charged NPs exhibited more punctate fluorescence associated with cells or tissues (Fig 12B). The negatively charged NPs seemed to transit the alimentary tract more rapidly than positively charged NPs. Indeed, negatively charged NPs were expelled and detected on the filter papers lining the plastic containers more frequently and abundantly (Fig 9). Negatively charged NPs were also detected more frequently in proboscis and head tissues of injected mosquitoes (Fig 10), suggesting that they trafficked more in the hemolymph than positively charged NPs. Whole body imaging analyses of parenterally challenged mosquitoes (Figs 2 and 3) revealed that negatively charged NPs exhibited greater MFI values and persisted longer in mosquitoes than positively charged NPs. It is noteworthy that the positively and negatively charged NPs exhibited the same phenotype in parenterally challenged larval mosquitoes (see Fig 5 in companion paper) [38]. In this regard, positively charged NPs are more efficiently internalized by mosquito cells (see Fig 1 in companion paper) [38]. Perhaps inefficient internalization of the negatively charged NPs conditions their persistence in the closed system of the hemocoel (Figs 2 and 3), which is in contrast to their rapid transit through the alimentary tract (Fig 9).

The preferred charge of NPs for environmental challenge of mosquitoes remains to be determined. Positively charged particles are more efficiently internalized in vector cells [38], but negatively charged NPs were detected abundantly in tissues in the proboscis, regardless of the mode of challenge. Cells in the labella of the proboscis of mosquitoes frequently contained or were associated with large accumulations of NPs (Figs 10B and 13). Sensory cells in the labella are of particular interest in terms of potential contact delivery of NPs. These organs are sampling the environment and could be a portal of entry of NP through oral or contact delivery of negatively charged NPs to control mosquitoes. In addition, negatively charged NPs more efficiently trafficked from the cuticle to mosquito organs than positively charged particles (Fig 13). Future studies will incorporate effector molecules in the NPs, which will then be used to challenge mosquitoes. Such studies will be most informative in selecting the optimal NPs for oral, contact, and parenteral delivery of effector molecules.

The large accumulation of NPs in the diverticula of mosquitoes is potentially important in terms of environmental delivery of NPs and their cargoes for vector control. Upon emergence, adult females typically ingest sugar meals to provide energy reserves for mating, host seeking, and other behaviors. Following ingestion, the sugar meal accumulates in the diverticula and is slowly released into the alimentary tract. The large NP load in the diverticula and their subsequent release into the alimentary tract provide ongoing opportunities for NP internalization by gut cells. Importantly, the sugar meal does not induce peritrophic matrix formation, which could serve as a barrier to NP contact with target cells. Sugar baited stations [44], which have proven to be very useful for arbovirus surveillance in mosquito populations, would thus be a potentially fruitful approach for delivering NPs and their cargoes to mosquitoes in nature.

Our results provide insights into NP design that could facilitate insect gene structure and function studies. The ability to deliver effector molecules through oral or contact challenge for gene structure function studies would be of great value and would preclude confounding effects of injection on gene regulation (e.g. induction of innate immune genes by penetration of the cuticle) and would also minimize mortality in experimental insects due to injection. Even with parenteral challenge, optimal internalization of NPs (e.g. positively charged particles) and delivery of their cargoes into target cells could greatly increase efficiency of gene silencing.

Our studies also provide important information for exploiting NP technology for development of new insecticides for mosquito vector control. Studies are in progress to define the preferred physicochemical properties of NPs for environmental delivery of effector molecules for gene silencing and vector lethality. The power of PRINT technology provides unparalleled capacity in this regard and for the development of a new generation of insecticides for insect vector and pest control.

## Supporting Information

S1 Fig Tissue tropisms and fluorescence intensity of positively and negatively charged 80 nm x 320 nm hydrogel nanoparticles (50 μg/mL) in *Anopheles gambiae* females following 1 day oral challenge. (A) Tissue tropisms (percent of mosquitoes with NP fluorescent signal of any intensity detected in the respective organ or tissue). (B) Fluorescence intensity (mean level of fluorescence intensity (NP load) in organs and tissues containing NPs) of positively and negatively charged 80 nm x 320 nm NPs. Tissue tropisms and fluorescence intensities in tissues were greatest at 1 or 2 d post challenge and then decreased dramatically. Fluorescence signal was not detected in organs or tissues in the head or proboscis indicating that the NPs were restricted to the alimentary tract. VD = ventral diverticulum; DD = dorsal diverticula; FG = foregut; MG = midgut; HG = hindgut; MT = Malpighian tubules; TM = thoracic muscles.
(TIF)

S2 Fig Tissue tropisms and fluorescence intensity of positively and negatively charged 200 nm x 200 nm hydrogel nanoparticles in *Anopheles gambiae* females following 1 day oral challenge. (A) Tissue tropisms (percent of mosquitoes with NP fluorescence signal of any intensity detected in the respective organ or tissue). (B) Flourescence intensity (mean level of fluorescence intensity (NP load) in organs and tissues containing NPs) of positively and negatively charged 200 nm x 200 nm NPs in *An*. *gambiae* following 1 day oral challenge with 250 μg/mL (A and B) or 50 μg/mL (C and D) of the respective NPs. Tissue tropisms and fluorescence intensities were greater and of longer duration when challenged with the higher dose of positively charged NPs. In contrast, for negatively charged NPs, the tissue tropisms and fluorescence intensities and duration were greater when mosquitoes were challenged with the lower dose. VD = ventral diverticulum; DD = dorsal diverticula; FG = foregut; MG = midgut; HG = hindgut; MT = Malpighian tubules; TM = thoracic muscles.
(TIF)

S3 Fig Tissue tropisms and fluorescence intensity of positively and negatively charged 80 nm x 5000 nm hydrogel nanoparticles in different tissues of *Anopheles gambiae* females following 1 day oral challenge. (A) Tissue tropisms (percent of mosquitoes with NP fluorescent signal of any intensity detected in the respective organ or tissue). (B) NP fluorescence intensity (mean level of fluorescence intensity (NP load) in organs and tissues containing NPs) of positively and negatively charged 80 nm x 5000 nm NPs in *An*. *gambiae* following 1 day oral challenge with 250 μg/mL (A and B) or 50 μg/mL (C and D) of the respective NPs. Tissue tropisms and flourescence intensities were greater and of longer duration when challenged with the higher dose of positively charged NPs. In contrast, for negatively charged NPs, the tissue tropisms and fluorescence intensities and duration were greater when mosquitoes were challenged with the lower dose. VD = ventral diverticulum; DD = dorsal diverticula; FG = foregut; MG = midgut; HG = hindgut; MT = Malpighian tubules; TM = thoracic muscles.
(TIF)

S4 Fig Detection of 80 nm x 320 nm hydrogel nanoparticles on filter papers in cages of *Anopheles gambiae* females following oral challenge. Small traces and spots of negatively charged NPs were detected on filter papers in the bottom of cages used for oral challenges of *Anopheles gambiae*. These may have resulted from tracking of NPs from the feeding chamber or from excreted NPs (Fig 9) on mosquito tarsi or on the proboscis of feeding mosquitoes. Bar = 1 μm.
(TIF)
