# Supplementary figures and images for: Biodistribution and Trafficking of Hydrogel Nanoparticles in Adult Mosquitoes
Source: PLoS Negl Trop Dis. 2015 May 21;9(5):e0003745. doi: 10.1371/journal.pntd.0003745 (PMC4440717; doi:10.1371/journal.pntd.0003745)

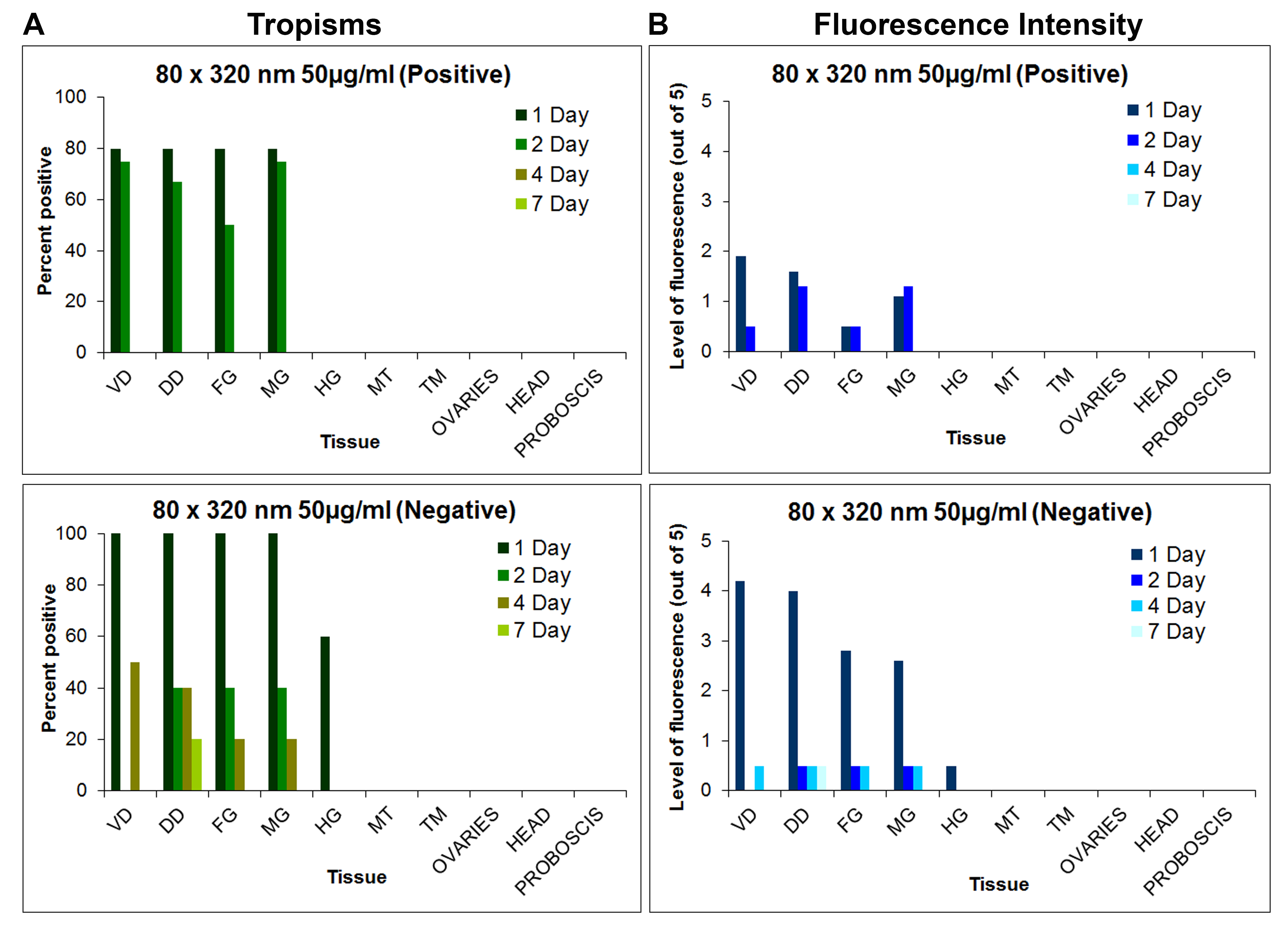

Supplement: S1 Fig — (A) Tissue tropisms (percent of mosquitoes with NP fluorescent signal of any intensity detected in the respective organ or tissue). (B) Fluorescence intensity (mean level of fluorescence intensity (NP load) in organs and tissues containing NPs) of positively and negatively charged 80 nm x 320 nm NPs. Tissue tropisms and fluorescence intensities in tissues were greatest at 1 or 2 d post challenge and then decreased dramatically. Fluorescence signal was not detected in organs or tissues in the head or proboscis indicating that the NPs were restricted to the alimentary tract. VD = ventral diverticulum; DD = dorsal diverticula; FG = foregut; MG = midgut; HG = hindgut; MT = Malpighian tubules; TM = thoracic muscles. (TIF) [file pntd.0003745.s001.tif]

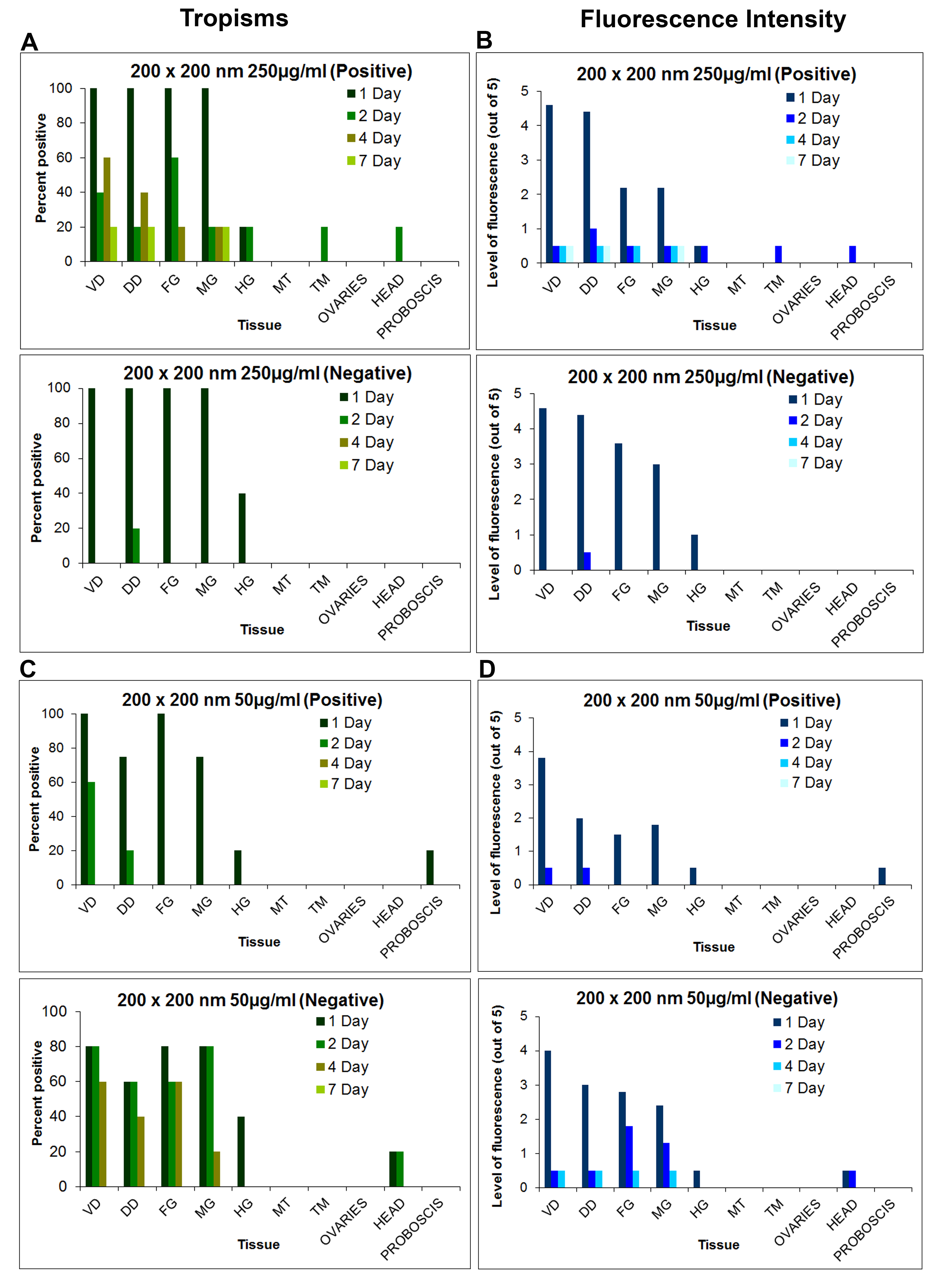

Supplement: S2 Fig — (A) Tissue tropisms (percent of mosquitoes with NP fluorescence signal of any intensity detected in the respective organ or tissue). (B) Flourescence intensity (mean level of fluorescence intensity (NP load) in organs and tissues containing NPs) of positively and negatively charged 200 nm x 200 nm NPs in An. gambiae following 1 day oral challenge with 250 μg/mL (A and B) or 50 μg/mL (C and D) of the respective NPs. Tissue tropisms and fluorescence intensities were greater and of longer duration when challenged with the higher dose of positively charged NPs. In contrast, for negatively charged NPs, the tissue tropisms and fluorescence intensities and duration were greater when mosquitoes were challenged with the lower dose. VD = ventral diverticulum; DD = dorsal diverticula; FG = foregut; MG = midgut; HG = hindgut; MT = Malpighian tubules; TM = thoracic muscles. (TIF) [file pntd.0003745.s002.tif]

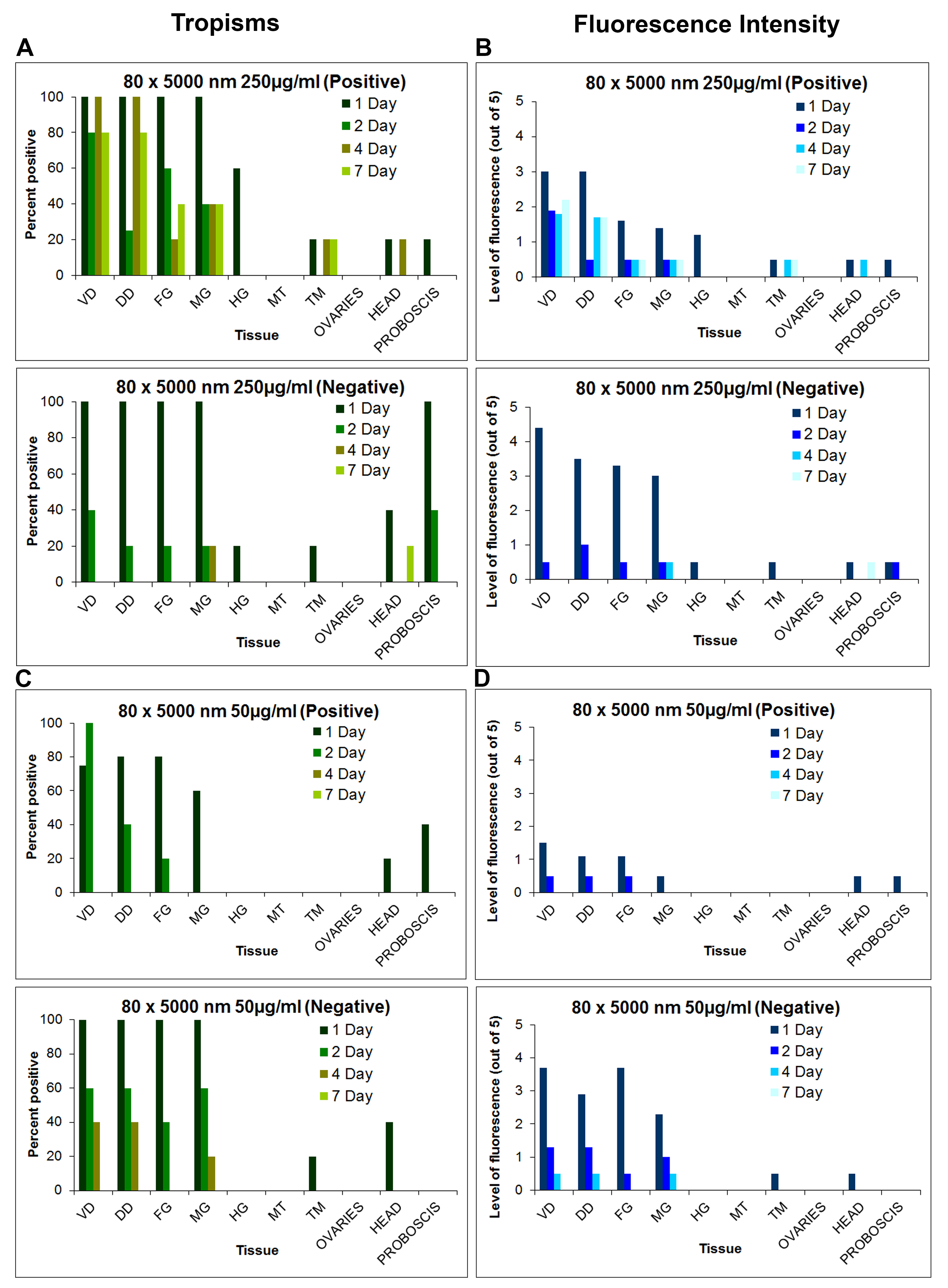

Supplement: S3 Fig — (A) Tissue tropisms (percent of mosquitoes with NP fluorescent signal of any intensity detected in the respective organ or tissue). (B) NP fluorescence intensity (mean level of fluorescence intensity (NP load) in organs and tissues containing NPs) of positively and negatively charged 80 nm x 5000 nm NPs in An. gambiae following 1 day oral challenge with 250 μg/mL (A and B) or 50 μg/mL (C and D) of the respective NPs. Tissue tropisms and flourescence intensities were greater and of longer duration when challenged with the higher dose of positively charged NPs. In contrast, for negatively charged NPs, the tissue tropisms and fluorescence intensities and duration were greater when mosquitoes were challenged with the lower dose. VD = ventral diverticulum; DD = dorsal diverticula; FG = foregut; MG = midgut; HG = hindgut; MT = Malpighian tubules; TM = thoracic muscles. (TIF) [file pntd.0003745.s003.tif]

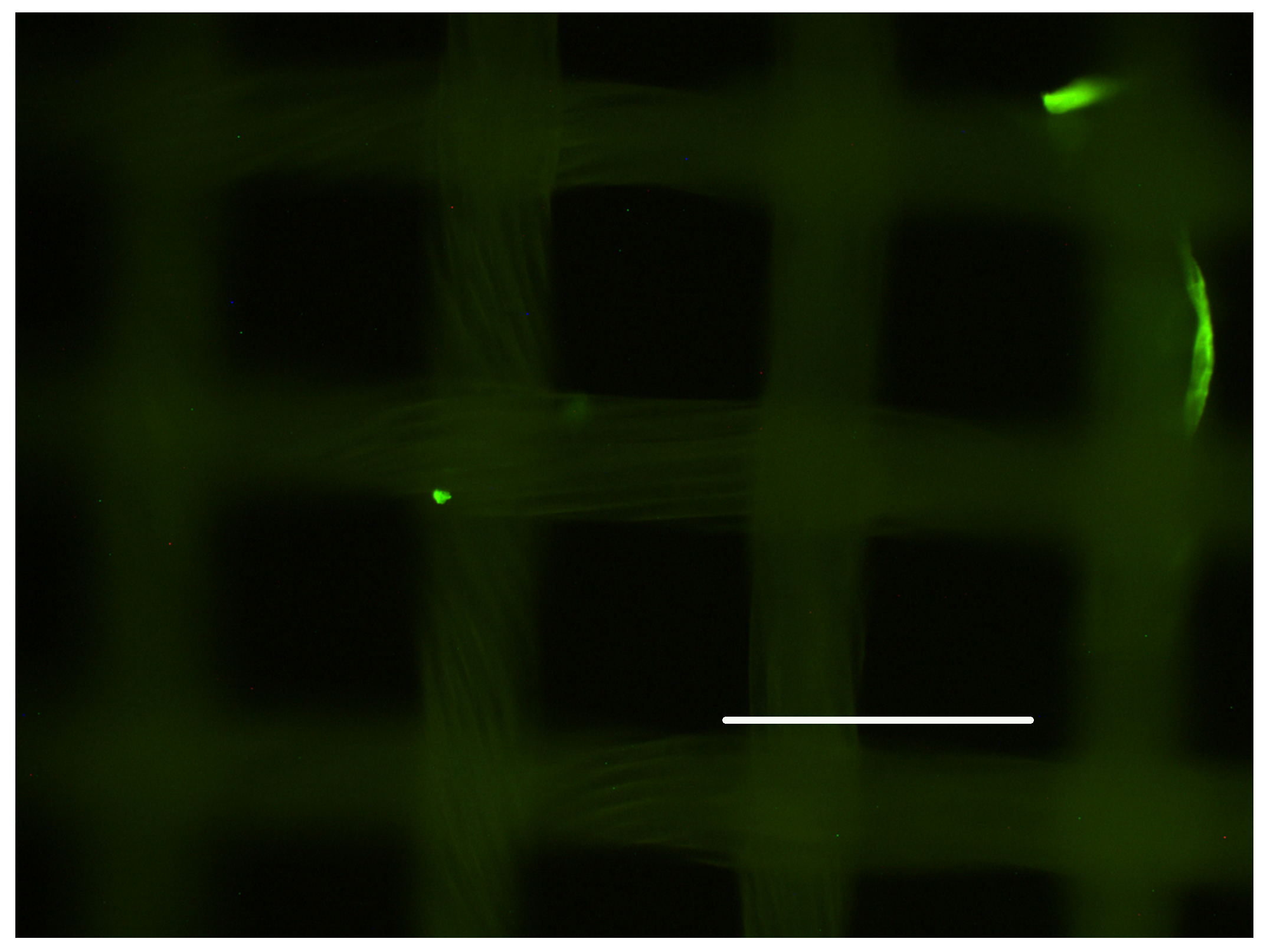

Supplement: S4 Fig — Small traces and spots of negatively charged NPs were detected on filter papers in the bottom of cages used for oral challenges of Anopheles gambiae. These may have resulted from tracking of NPs from the feeding chamber or from excreted NPs (Fig 9) on mosquito tarsi or on the proboscis of feeding mosquitoes. Bar = 1 μm. (TIF) [file pntd.0003745.s004.tif]
